# The Effect of C-Arm Mobility and Field of Vision on Radiation Exposure in the Treatment of Proximal Femoral Fractures: A Randomized Clinical Trial

**DOI:** 10.1155/2018/6768272

**Published:** 2018-03-27

**Authors:** Mahmut Kalem, Kerem Başarır, Hakan Kocaoğlu, Ercan Şahin, Hakan Kınık

**Affiliations:** ^1^Department of Orthopedics and Traumatology, Ankara University Faculty of Medicine, Ankara, Turkey; ^2^Department of Orthopedics and Traumatology, Bulent Ecevit University Faculty of Medicine, Zonguldak, Turkey

## Abstract

**Objectives:**

To examine the effect of fluoroscopy devices with different sizes of image intensifier and C-arm maneuverability on operating time, fluoroscopy time, radiation dose and reduction, and fixation quality at intertrochanteric femoral fractures.

**Design:**

Single-center, randomized, prospective study.

**Setting:**

Academic Level I trauma hospital.

**Patients and Intervention:**

34 patients treated with cephalomedullary nailing for a stable, intertrochanteric proximal femur fracture (OTA A1).

**Main Outcome Measurement:**

The total working time of the fluoroscopy device, the dose-area product (DAP), operating time, reduction quality (cortical continuity, symmetrical collodiaphyseal angle, and shortness), and fixation quality (Bosworth quadrants, the tip-apex distance, TAD).

**Results:**

There were no cases of poor reduction; also the placement of the blade was optimal for 14 patients and suboptimal in 3 patients in each group. Superior-posterior placement of the blade or TAD > 25 mm was not seen in any patient. Total operating time was significantly shorter when using device A compared to the use of device B (20.1 ± 3.4 mins versus 25.3 ± 5.4 mins, *p* < 0.001). Total radiation time was significantly shorter with device A compared to the use of device B (58.1 ± 19.4 secs versus 98.9  ±  55.4 secs, *p* = 0.008). The measured radiation dose was lower with the use of device A compared to device B (3.5  ±  1.2 Gy·cm^2^ versus 7.3  ±  4.5 Gy·cm^2^, *p* = 0.002).

**Conclusion:**

Physical properties of fluoroscopy devices used during the fixation of intertrochanteric fractures could yield significant differences in operating times and the radiation dose while having comparable clinical results.

## 1. Introduction 

The use of fluoroscopy for guidance in orthopedic trauma surgery has significantly increased, allowing for smaller surgical exposures to achieve reductions and internal fixation of fractures [1–4]. Over the years, there has been a tremendous increase in the use of fluoroscopy in orthopedic surgery, especially in treating proximal femur fractures [5, 6].

According to the aim for which current fluoroscopy devices are used, there are differences in software, power, working distance, maneuver capability of the C-arm, and image intensifier size. The basic principle in reducing radiation exposure caused by fluoroscopy devices is to keep the ionized radiation dose as low as possible [7, 8]. In literature it has been reported that many parameters are known to affect operating time, fluoroscopy time, and the consequent ionized radiation dose. It is thought that one of these could be the technical property of the fluoroscopy device. Although the effects of fluoroscopy devices with different properties on operating time and radiation exposure have been researched in orthopedic surgery, the effects on the outcomes of their use in proximal femur fracture percutaneous treatment are not known [1, 9–12]. This study was undertaken with the thought that high C-arm maneuver capability and a wide field of vision could have an effect on operating time and exposure to ionized radiation. Therefore, the aim of the study was to examine the effect on operating time, fluoroscopy time, radiation dose, and reduction and fixation quality of 2 fluoroscopy devices of the same make and with the same software but with different sizes of image intensifier, which affects the field of vision, and different C-arm maneuver capability, which facilitates use during surgery; the devices were used by the same surgeon in the treatment of stable intertrochanteric femoral fractures using single fixation device.

## 2. Patients and Method

This single-center, prospective study included 34 patients treated with cephalomedullary nailing for a stable, intertrochanteric proximal femur fracture (Orthopaedic Trauma Association [OTA] A1 classification). Approval for the study was granted by the Local Ethics Committee. Between December 2016 and March 2017, a total of 134 consecutive patients presented at the Emergency Department of a major trauma center following a fall and were diagnosed with intertrochanteric femoral fracture and were treated with closed reduction and fixation with cephalomedullary nailing. Demographic characteristics (age, gender, side, and body mass index) were recorded as well as data related to the fracture type, operating time, the fluoroscopy device used, fluoroscopy time, radiation dose and reduction, and fixation quality. Patients with an AO/OTA Type A1 (stable) fracture and age > 60 years were included in the study. Patients with an additional fracture or pathological fracture were excluded from the study. Informed consent was obtained from all the patients or from a first-degree relative in the case of patients with dementia. All patients were operated on within 48 hours of arrival at the hospital.

**Figure 1:**
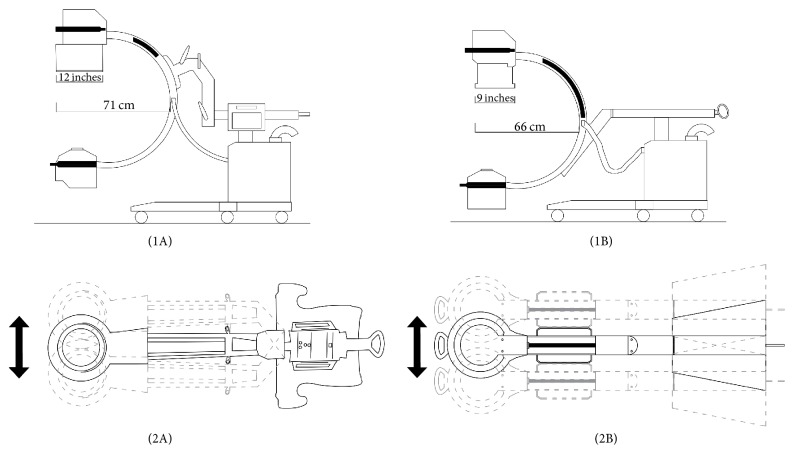
The distinct features of two fluoroscopy devices used (device A,* OEC 9900 Elite, General Electric Healthcare, USA*; device B,* OEC Brivo 785 Essential, General Electric Healthcare, USA*). ((1A) and (1B)) Device A having a bigger image intensifier size and longer C-arm depth, which affects the field of vision, when compared to device B. ((2A) and (2B)) Device A has more degree of freedom of C-arm maneuverability when compared to device B. This enables the technician to find the area of interest only moving the C-arm while keeping the device still thus facilitating the use during surgery.

**Figure 2:**
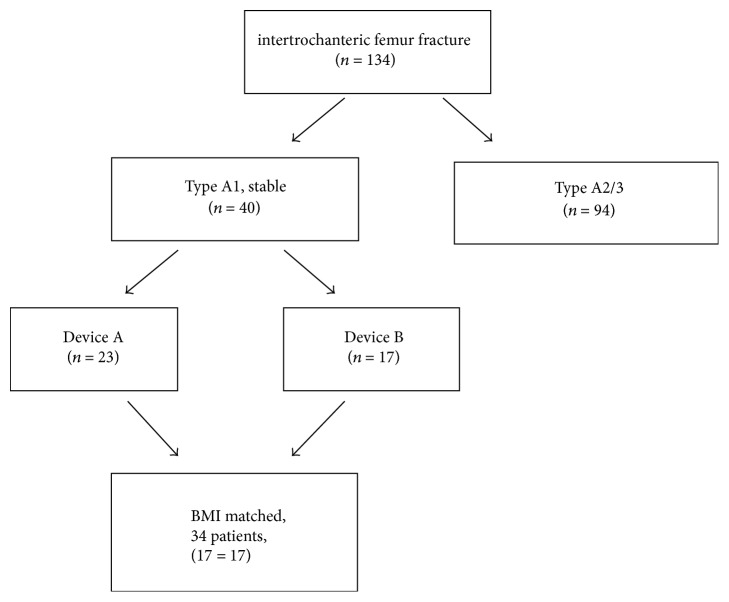
Scheme explaining patient selection process.

From the total 134 patients, 40 were A1 stable intertrochanteric fractures. Randomization was achieved by the availability of the devices, when the patient was operated on. 17 patients were operated on with device B* (OEC Brivo 785 Essential, General Electric Healthcare, USA) *on days when device A* (OEC® 9900 Elite, General Electric Healthcare, USA)*, which has the property of being able to apply digital subtraction angiography (DSA), was used by the vascular surgeons and 23 patients were operated on with device A in the orthopedic operating theatre on days when it was available. These two fluoroscopy devices were of the same make and with the same software but with different sizes of image intensifier, which affects the field of vision, and different C-arm maneuver capability, which facilitates use during surgery (Figure 1). Patients operated on using device A were BMI matched to the 17 patients of device B and thus the 2 study groups were formed (Figure 2).

All the patients were given regional anesthesia and were operated on in a supine position on a radiolucent table with the fractured hip and whole extremity draped. The fluoroscopy device was set up on the opposite side in a manner to show the fracture region. Fracture reduction and protection were provided with manual traction only by a single assistant. Reduction evaluation and the fixation application were made under fluoroscopy guidance. All the operations were performed by a single surgeon (MK). In all patients, internal fixation was applied using cephalomedullary nailing (*standard proximal femoral nail antirotation,* PFNA; Synthes GmbH, Oberdorf, Switzerland). The fluoroscopy device in automatic dose control mode was used by the same radiology technician in all cases.

The total working time of the fluoroscopy device during the surgical procedure and the radiation dose used were obtained from the records of the fluoroscopy device at the end of surgery. The dose-area product (DAP) recorded during fluoroscopy imaging was the measurement method of the radiation dose selected in this study. DAP is a quantity used in assessing the radiation risk from radiographic examinations and interventional procedures and is defined as the absorbed dose multiplied by the area irradiated [6, 11, 13]. The operating time was calculated as the time from the initial incision to closure of the wound.

All the radiographic measurements were made by one of the authors (ES, who is blinded to the surgical intervention) on the standard AP as well as lateral radiographs in internal rotation obtained at the end of surgery, using Centricity PACS-IW software (General Electric Healthcare). Reduction quality was classified as described by Schipper et al. as anatomic (cortical continuity, symmetrical collodiaphyseal angle, and no shortness), good (5°–10° varus/valgus), and poor (>10° varus/valgus) [14]. For fixation quality, the Cleveland-Bosworth quadrants were used which evaluate the position of the neck screw within the femoral head and the tip-apex distance (TAD) was measured [15]. Optimal fixation was accepted as central-central and inferior-central placement of the blade and TAD < 25 mm. Placements outside of this were accepted as suboptimal fixation, with the worst being a superior-posterior placement.

Statistical evaluations were made using IBM SPSS 15 (SPSS Inc., Chicago, IL, USA) software. Conformity of the data to normal distribution was evaluated with the Shapiro-Wilk test. Numerical variables were stated as mean ± standard deviation (SD) and categorical variables as percentages (%). Differences between the groups for numerical variables not with normal distribution were examined with the Mann–Whitney *U* test. A value of *p* < 0.05 was accepted as statistically significant.

## 3. Results

The study included a total of 34 patients comprising 17 males and 17 females with a mean age of 77.8  ±  9.0 years in the device A group and 77.8  ±  12.4 years in the device B group (*p* > 0.05) (Table 1).

When the radiological results were evaluated, in the device B group, anatomic reduction was obtained in 15 patients (88.2%) and good reduction in 2 (11.8%). In the device A group, anatomic reduction was obtained in all 17 patients (100%). There were no cases of poor reduction in either group (*p* > 0.05) (Table 2).

When the fixation quality was evaluated, optimal placement of the blade was the same in both groups (*n* = 14, 82.3%). Suboptimal placement was observed in 3 patients in each group. Superior-posterior placement of the blade was not seen in any patient. In both groups, the TAD measurement was <25 mm in all patients (Table 2).

**Figure 3:**
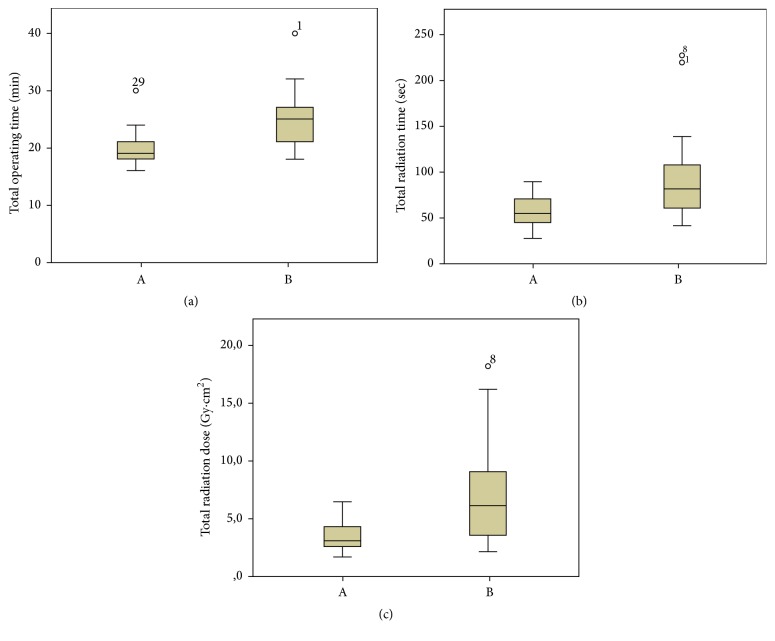
Box-plot graphs showing the results. (a) Box-plot showing total operation time for device A and device B. (b) Box-plot showing total radiation time for device A and device B. (c) Box-plot showing total radiation dose for device A and device B.

Total operating time was significantly shorter when using device A compared to the use of device B (20.1 ± 3.4 mins versus 25.3 ± 5.4 mins, *p* < 0.001). Total radiation time was significantly shorter with device A compared to the use of device B (58.1 ± 19.4 secs versus 98.9 ± 55.4 secs, *p* = 0.008). The measured radiation dose was lower with the use of device A compared to device B (3.5  ±  1.2 Gy·cm^2^ versus 7.3  ±  4.5 Gy·cm^2^, *p* = 0.002) (Figure 3).

## 4. Discussion

The functional results following treatment of hip fractures with percutaneous methods are closely related to reduction and fixation quality and the most important factor in achieving this is obtaining a good quality field of vision during the surgical procedure [10]. However, Mastrangelo et al. stated the importance of exposure to radiation caused during imaging and reported that orthopedic surgeons were exposed to 4 times more radiation than other surgeons and 8 times more than other healthcare personnel and there were increased rates of cancer in those working in orthopedics [16]. The amount of ionized radiation that occurs during imaging is known to be associated with the exposure time, the distance of the fluoroscopy device from the patient, the BMI of the patient, the projection angle and the extremity level, the type of operating table, and the experience of the surgeon [5, 12, 13, 17]. The results of the current study revealed that the technical properties of the fluoroscopy device had a significant effect on operating time, fluoroscopy time, and the amount of ionized radiation. To discount variables other than the fluoroscopy device in this study, all the surgical procedures were performed by the same surgeon; the same fixation system was used with the consideration that there may have been an effect of differences created in the application of different systems, and only AO/OTA A1 fractures were examined which could be reduced with manual traction only taking into consideration the effects that could have arisen from direct reduction methods.

The operating time for the treatment of intertrochanteric femoral fractures with closed reduction and nailing has been reported in literature at varying periods in the range of 20 mins to 67 mins [18–21]. The reasons for such differences in the measurement of operating time can be considered to be that studies have been made on different types of fractures; the period has included different surgical steps, the type of anesthesia, the use of manual traction or a traction table, the implant selection, and the experience of the surgeon. When the operating times of the current study are compared with those in literature, they are consistent and a significant difference was determined between the groups (device A: 20.1 ± 3.4 min; device B: 25.3 ± 5.4 min, *p* < 0.001).

The fluoroscopy time in the closed reduction and nailing of intertrochanteric fractures has been associated with factors such as the severity of the fracture, implant selection, patient position, and the experience of the surgeon and radiology technician [5, 22–25]. There has been mention in literature of fluoroscopy times in trochanteric fractures with PFNA fixation but there are insufficient data on the use in stable A1 fractures. In a study by Zehir et al., the application of PFNA was evaluated in 92 patients, most of whom had an A2 fracture, and fluoroscopy time was reported as a mean of 1.50 min [26]. The fluoroscopy times in the current study were consistent with those in literature and a statistically significant difference was determined between the groups (device A: 58.1 ± 19.4 secs; device B: 98.9 ± 55.4 secs, *p* = 0.008).

When the radiation doses in the treatment of intertrochanteric fractures with cephalomedullary nailing are evaluated in literature, the DAP measurements associated with the fluoroscopy times are between 0.8 and 11 Gy·cm^2^ [27–29]. The differences in radiation dose are likely associated with surgical experience and competence and condition of surgical sites [27]. The DAP measurements in the current study are consistent with literature and a statistically significant difference was determined between the groups (device A: 3.5 ± 1.2 Gy·cm^2^; device B: 7.3 ± 4.5 Gy·cm^2^, *p* = 0.002).

To acquire images in a different plane, changing the place of the fluoroscopy device takes a certain time and extra exposures during the procedure [2, 10]. Even there are two distinct papers in the literature that argue the use of two C-arms simultaneously to face these problems [10, 30]. In the current study, the significant difference between the groups in respect of the operating times is thought to be due to the difference in the C-arm maneuver capability. Due to the larger image intensifier it was possible to take anterior-posterior and lateral images of the fracture region of interest in a single shoot and this was considered to be a reason for the significant differences in fluoroscopy times and therefore the doses of radiation exposure.

The most important limitation of the study was that the power analysis results were not sufficient as the number of patients was low. Future studies examining more extensive series would be beneficial in establishing which parameters directly affect operating time, fluoroscopy time, and the amount of ionized radiation.

## 5. Conclusion

The results of this study showed that different physical properties of fluoroscopy devices used during the fixation of intertrochanteric fractures with cephalomedullary nailing caused differences in operating times and the radiation dose without having any effect on the radiological results. It can be considered appropriate to make the selection of a fluoroscopy device taking into account the frequency of application and cost.

## Figures and Tables

**Table 1:** The demographic distribution of patients.

Variable	Device A (*N* = 17)	Device B (*N* = 17)
Age (yr)	77,8 ± 9,0	77,8 ± 12,4
Gender		
Female	10 (58,8%)	7 (41,2%)
Male	7 (41,2%)	10 (58,8%)
Laterality		
Left	9 (52,9%)	7 (41,2%)
Right	8 (47,1%)	10 (58,8%)

**Table 2:** The demographic radiological results of patient.

	Device A (*N* = 17)	Device B (*N* = 17)
Reduction		
Anatomic	17 (100%)	15 (88,2%)
Good	-	2 (11,8)
Bad	-	-
Blade position		
Optimal	14 (82,3%)	14 (82,3%)
Suboptimal	3 (17,7%)	3 (17,7%)
TAD (mm)	19,4 ± 2,9	22,0 ± 3,8
